# Massive pulmonary embolism in patients with extreme bleeding risk: a case series on the successful use of ultrasound-assisted, catheter directed thrombolysis in a district general hospital

**DOI:** 10.1007/s11239-020-02258-6

**Published:** 2020-09-04

**Authors:** Jingzhou He, Benjamin Clayton, Hibba Kurdi, Michael Gibbons, Anthony Watkinson, Andrew S. P. Sharp

**Affiliations:** 1 Department of Cardiology, Royal Devon and Exeter NHS Foundation Trust, Barrack Road, Exeter, EX2 5DW UK; 2 Diabetes and Vascular Medicine Research Centre, University of Exeter, Barrack Road, Exeter, EX2 5AX UK; 3 Department of Cardiology, Morriston Cardiac Center, Swansea, SA6 6NL UK; 4 Department of Respiratory Medicine, Royal Devon and Exeter NHS Foundation Trust, Barrack Road, Exeter, EX2 5DW UK; 5 Department of Interventional Radiology, Royal Devon and Exeter NHS Foundation Trust, Barrack Road, Exeter, EX2 5DW UK; 6 Department of Cardiology, University Hospital of Wales, Heath Park Way, Cardiff, CF14 4XW UK

**Keywords:** Massive Pulmonary Embolism, Thrombolysis, Catheter directed thrombolysis, Interventional cardiology, Bleed risk

## Abstract

Massive pulmonary embolism (PE), characterised by profound arterial hypotension, is a life-threatening emergency with a 90-day mortality of over 50%. Systemic thrombolysis can significantly reduce the risk of death or cardiovascular collapse in these patients, by around 50%, but these benefits are offset by a fivefold increased risk of intracranial haemorrhage and major bleeding, which may limit its use in patients at high risk of catastrophic haemorrhage. We describe a case series of 3 patients presenting with massive PE, each with extreme risk of bleeding and contra-indication to systemic thrombolysis, treated successfully with ultrasound-assisted, catheter directed thrombolysis (U-ACDT). Our experience of this novel technique using the EkoSonic Endovascular System (Ekos, BTG, London, UK) on carefully selected patients has demonstrated the potential to improve clinical status in shocked patients, with minimal bleed risk. There have been several clinical studies evaluating the Ekos system. Both the ULTIMA and SEATTLE II studies have shown significant reductions in RV/LV ratio by CT scanning when compared to standard anticoagulation in patients with intermediate-risk PE, with minimal bleeding complications. However, there is a pressing need for a randomised trial demonstrating improvement in robust clinical outcomes when comparing U-ACDT to simple anticoagulation. We believe that this case series adds new insight and highlights the potential of catheter directed thrombolysis in this high-risk patient cohort and consideration should be made to its use in cases where systemic thrombolysis is felt to be too high risk.

## Highlights

- Management of multi-comorbid patients with life-threatening massive PE is complex, and competent multidisciplinary PERT input in decision-making is vital.
- These cases have shown the potential of U-ACDT in patients with massive PE and extreme bleed risk with good outcomes.
- Further comprehensive, prospective and randomised evaluation is required in both patients with submassive, and massive PE.

## Summary

Massive pulmonary embolism (PE), characterised by profound arterial hypotension, is a life-threatening emergency with a 90-day mortality of over 50% [1]. Systemic thrombolysis can significantly reduce the risk of death or cardiovascular collapse in these patients, by around 50%, but these benefits are offset by a fivefold increased risk of intracranial haemorrhage and major bleeding, which may limit its use in patients at high risk of catastrophic haemorrhage [1–3].

Subsequently, advanced novel technologies have been developed with lower bleed risk. We describe the effective co-ordination of our multidisciplinary pulmonary embolism response team (PERT) and successful treatment of three patients with massive PE and extreme bleeding risk with ultrasound-assisted, catheter directed thrombolysis (U-ACDT).

## Methods

The EkoSonic Endovascular System (BTG, London, United Kingdom) uses a catheter to deliver thrombolytic directly to pulmonary arteries. Ultrasound emitters emit high frequency, low power ultrasound, intended to disaggregate fibrin fibres and thereby improve drug delivery, allowing use of a fraction of the systemic fibrinolytic dose [4].

Patients were selected based on clinical need at a single institution (Royal Devon & Exeter Hospital, Exeter, UK) without on-site cardiothoracic surgery, having had a confirmed diagnosis with CT pulmonary angiography (CTPA) and a physiological assessment with echocardiography. Each case was discussed by multidisciplinary team (comprising cardiology, respiratory, intensive care, and interventional radiology) and the need for emergency treatment confirmed, along with the contraindications to systemic thrombolysis. Subsequently, cases were discussed with our institution’s medical director or his immediate deputy to approve use.

All patients were transferred to the cardiac catheter laboratory at our institution, with haemodynamic monitoring throughout. Under aseptic conditions, two 6F sheaths were inserted with the use of a 4F micro-puncture needle under direct ultrasound guidance into the right femoral vein. A long 0.035 guidewire and multipurpose catheter were used to traverse the right heart into the pulmonary arteries. An ultrasound catheter was exchanged over the wire and inserted into the left, right, or both pulmonary arteries (depending on thrombus burden). Alteplase was infused at a rate of 1 mg/catheter/h along with saline coolant at 35 mL/h, with a fixed infusion of unfractionated heparin (500 iu/h) into a peripheral vein. Echocardiography was performed immediately post procedure, at 6 h, and again the following day.

### Patient 1

**Fig. 1:**
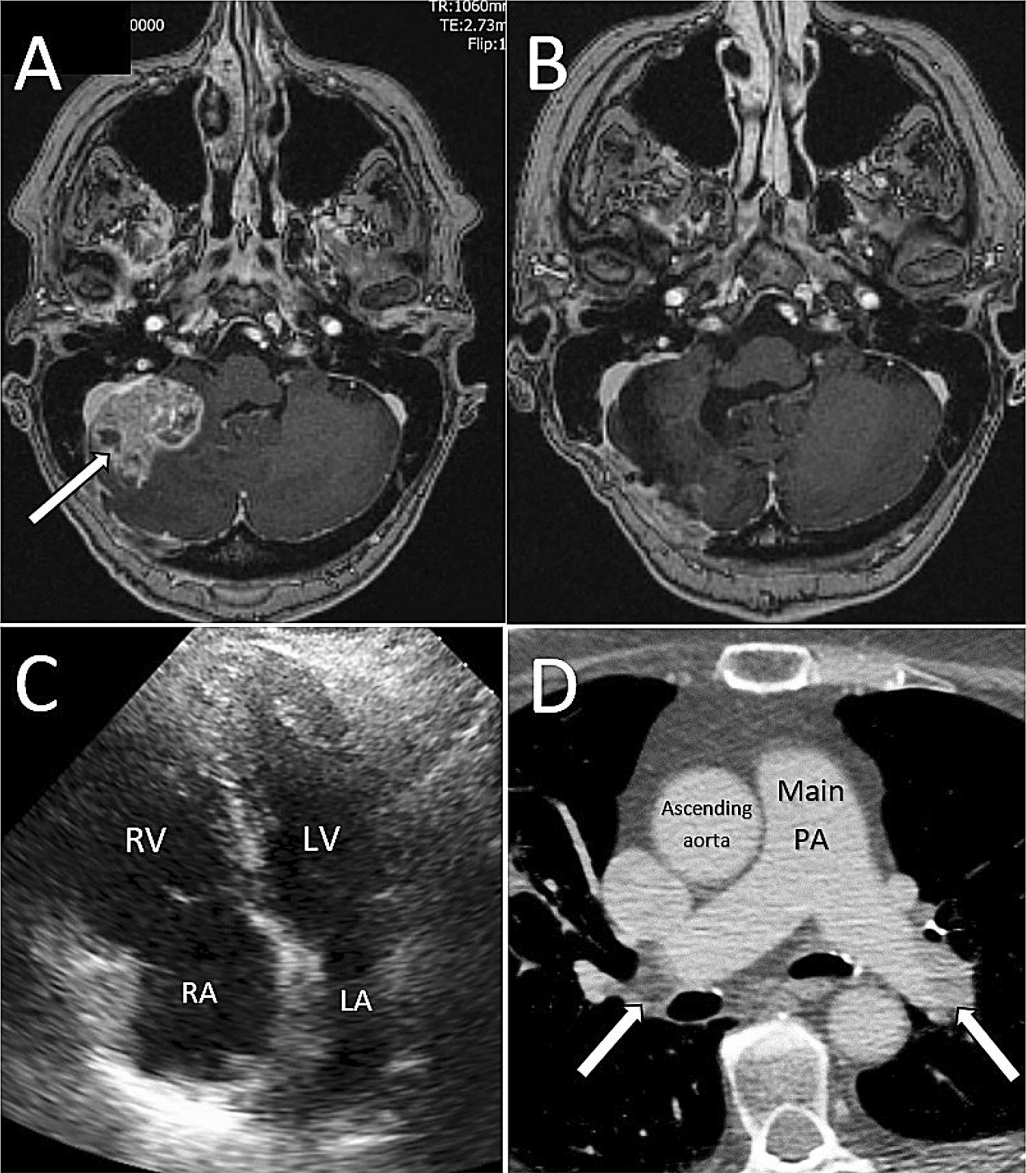
**a** Right sided solitary cerebellar metastasis pre, and **b** post-surgery, **c** Echocardiogram showing dilated right ventricle, **d** CTPA with gadolinium contrast showing bilateral PE

A 73-year-old lady was admitted with abrupt circulatory collapse, with an arterial blood pressure of 55/35 mmHg 7 days after undergoing retro-mastoid craniectomy and excision of a solitary cerebellar metastasis (Fig. 1a pre, 1b post-surgery) secondary to treatment-responsive adenocarcinoma of the lung. Her ECG showed widespread, non-specific T-wave changes and bedside echocardiography demonstrated severe right heart dilatation and impairment, with septal flattening (Fig. 1c).

Emergency CTPA, performed using gadolinium due to a history of anaphylaxis to iodinated contrast, confirmed the diagnosis of massive PE with bilateral proximal thrombus (Fig. 1d). Her pulmonary embolism severity index (PESI) was calculated at 273 [5].

After MDT discussion, where systemic thrombolysis was felt to be too high risk of intra-cranial haemorrhage, she was transferred to the cardiac catheter lab, requiring intravenous adrenaline both *en route* and repeatedly during the case for profound hypotension. U-ACDT was performed to both pulmonary arteries. At five hours, following 9 mg of alteplase, normal haemodynamic parameters had been restored. She returned to the rehabilitation ward at 48 h, and was discharged home 8 days later, at Cerebral Performance Category 1 [6], with persistence of post-operative diplopia, but no new neurological impairment.

### Patient 2

A 70-year-old hypertensive female presented with syncope following 2 days of dyspnoea, chest pain and pre-syncope, on a background of 1 week of right iliac fossa pain. She was transported via air ambulance and on arrival in the emergency department was hypotensive, with a blood pressure of 90/50 mmHg, and hypoxic, with peripheral saturations of 83% on air.

**Fig. 2:**
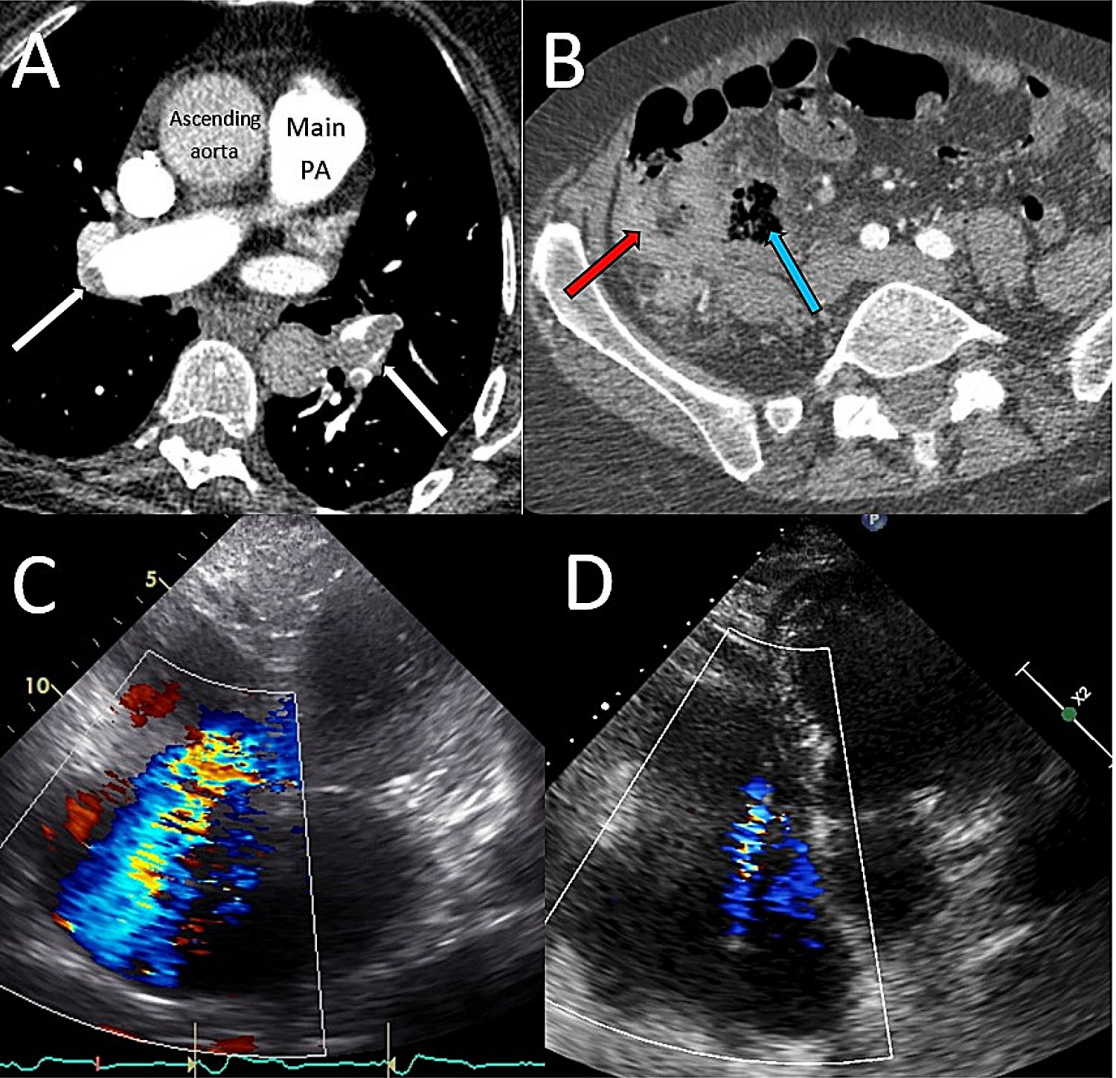
**a** CTPA showing bilateral PE, **b** CT abdomen showing marked caecal thickening (red arrow) and adjacent air pocket secondary to bowel perforation (blue arrow), **c** and **d** degree of TR pre, and post U-ACD

A point of care haemoglobin was measured at 70 g/L. She underwent urgent CTPA, with coverage extended to include the abdomen given her clinical history. This demonstrated extensive bilateral PE (Fig. 2a) with right heart dilatation and marked caecal thickening, suggestive of malignancy, with localised bowel perforation (Fig. 2b).

Bedside echocardiography confirmed severe right heart dilatation and impairment, with pulmonary hypertension (RV:LV 1.8, estimated pulmonary artery systolic pressure (PASP = 50 mmHg, severe tricuspid regurgitation—Fig. 2c). Her high-sensitivity troponin T was elevated at seven times the normal upper limit.

She was commenced on broad spectrum antibiotics and reviewed by the PERT team. It was clear she was unlikely to survive without early bowel surgery, which could not take place given her haemodynamic instability. Systemic thrombolysis was felt to be too high risk for intra-abdominal haemorrhage.

She therefore underwent U-ACDT in the cardiac catheter laboratory, using a total of 9 mg alteplase over 4.5 h without any exacerbation of her chronic gastrointestinal haemorrhage. The following day, her blood pressure had improved to 135/75 mmHg. There was improvement in her right ventricular and right atrial dimensions, reduction in the PASP and improvement in the tricuspid regurgitation (Fig. 2d).

Two weeks later she underwent a right hemicolectomy, with excision of a large caecal tumour involving the ovary and small bowel mesentery. After 36 h on the intensive care unit (ICU), she was returned to the ward, and was discharged home 8 days later.

### Patient 3

A 33-year-old woman admitted for an elective Caesarean section for her second pregnancy suffered a cardiac arrest shortly after anaesthetic induction. The baby was successfully delivered, and cardiopulmonary resuscitation achieved return of spontaneous circulation. She was transferred to the ICU where she became haemodynamically unstable. She was taken back to theatre where ongoing bleeding was identified, and an emergency hysterectomy was performed.

**Fig. 3:**
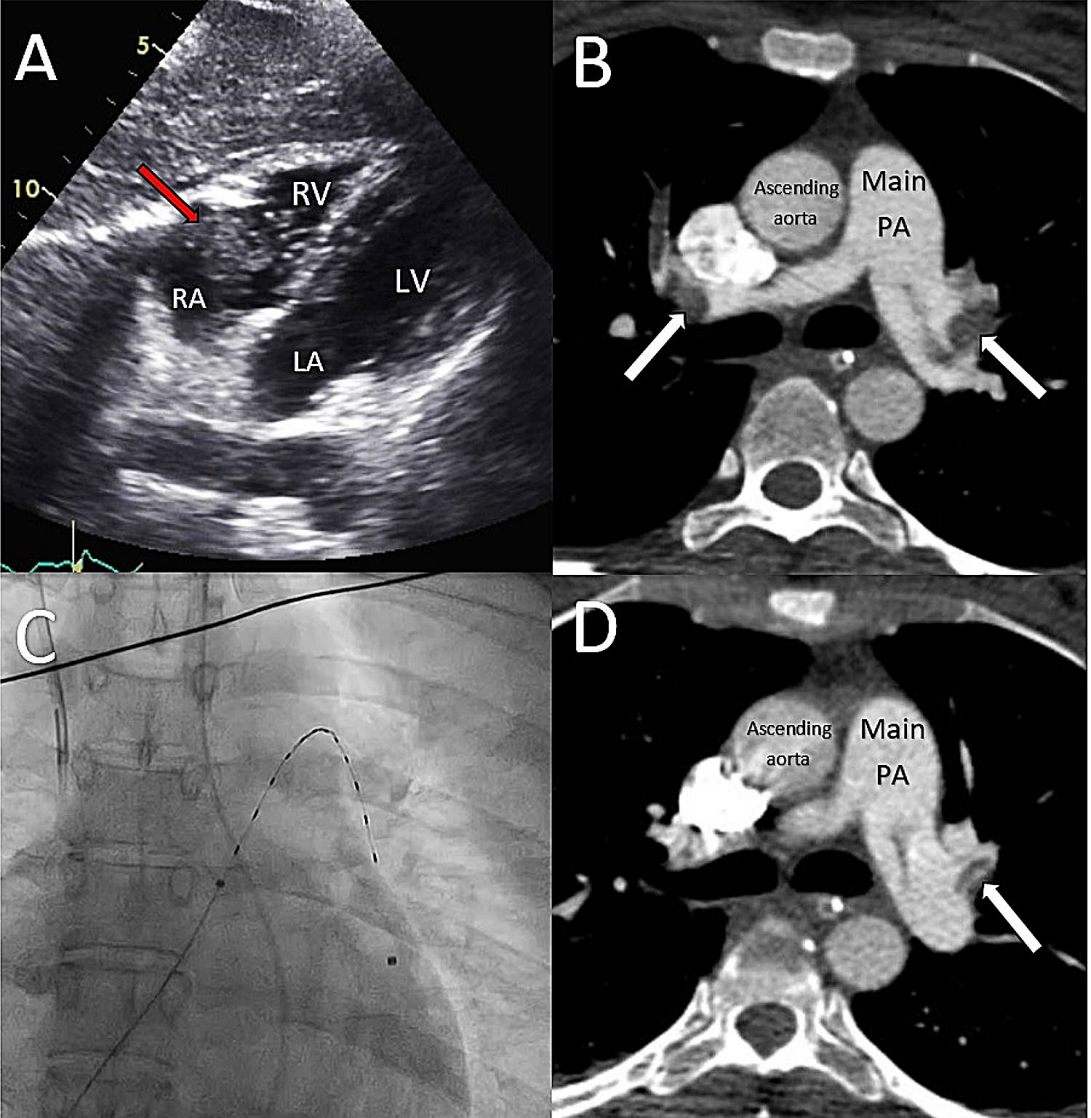
**a** Transthoracic echocardiogram from the subcostal view showing large RA thrombus, **b** CTPA showing bilateral PE, **c** EKOS catheter in left PA, **d** repeat CTPA 3 days later showing reduced clot burden in left PA

On return to ICU, she suffered a further cardiac arrest. Emergency transthoracic echocardiography demonstrated a dilated and impaired right heart and a large right atrial thrombus (Fig. 3a). Given her major abdominal surgery and bleeding risk, she was initially managed conservatively with intravenous heparin, requiring intermittent vasopressor support. Repeat echocardiography at 3 h showed the absence of right atrium thrombus, presumably due to embolisation.

On day 1 post-surgery, she became increasingly unstable, with progressive hypotension, rising lactate and coagulopathy (INR 2.8, APTR 2.0, fibrinogen undetectable) and thrombocytopenia. CTPA showed a saddle PE with heavy clot burden particularly in the left pulmonary artery (Fig. 3b), dilated right heart and a RV:LV ratio of 1.2. On day 2, following multi-disciplinary review, and with increasing inotrope requirements, a decision was made to attempt U-ACT.

In the cardiac catheter laboratory, a single ultrasound catheter was inserted into the left pulmonary artery (Fig. 3c). A total of 14 mg of alteplase was infused over 24 h through the device. During this time, she experienced no bleeding complications. At the end of the procedure all inotropic support was able to be withdrawn and her PASP was 19 mmHg.

Repeat CTPA 3 days later showed a significantly reduced clot burden in the left pulmonary artery with a reduction in size of the right ventricle (Fig. 3d). She was extubated on day 5, mobilising around the ward on day 8 and discharged on day 10. At 1 year, both patient and baby are well, and the patient’s echocardiogram demonstrates normal right ventricular size, function and estimated pulmonary artery pressures.

## Discussion

Venous thromboembolism (VTE) is the third most common cardiovascular disease, with an annual incidence of between 75 and 269 cases per 100 000 [5]. Most cases (60%) are low risk and managed with oral anticoagulation with a low risk of death (approx. 2%). This increases to 30% in the presence of shock and as high as 70% if cardiac arrest occurs [6]. In patients with massive PE with haemodynamic collapse and shock, full-dose systemic thrombolysis is required as a salvage intervention.

In these critically unwell patients, the importance of timely formulation of effective treatment by specialist is vital. This is reflected by the emergence of multidisciplinary rapid-response teams for the treatment of intermediate/high risk PE. The set-up of PERT is encouraged by the latest 2019 ESC and European Respiratory Society PE guidelines [7].

### Conventional lysis

The use of systemic thrombolysis is associated with a significant reduction in mortality—approximately half compared to anticoagulation alone, even with intermediate risk PE, but associated with a significant risk of major bleeding [2, 3, 8].

It is therefore unsurprising that most contraindications to thrombolysis are related to bleeding risk. The American Heart Association (AHA) and European Society of Cardiology (ESC) guidelines are similar, with absolute contraindications including known malignant intracranial neoplasm or recent surgery (within 3 weeks) encroaching on the spinal canal or brain, and patients with active (or at high risk of) bleeding [9, 10]. The ESC also considers gastrointestinal bleeding within 4 weeks an absolute contraindication, and pregnancy as a relative contraindication. However, the ESC guideline concludes that “most contraindications to thrombolysis should be considered relative in patients with life-threatening, high-risk PE.”[9].

Unfortunately, the aetiology of PE often includes contraindication(s) to its treatment. Malignancy is a well-recognised risk factor for the development of pulmonary emboli, conferring a relative risk of up to 7 times that of the general population, with brain tumours associated with particularly high risk [11, 12]. Pregnancy is another high-risk state, with a sixfold incidence in PE [13]. The development of PE is also more likely in patients who are convalescing due to intercurrent illness, which may also preclude conventional thrombolysis. Age is also highly relevant, with those over the age of 65 at greatest risk from lytic therapy [2, 8].

### Catheter-based techniques

Historically, there have been limited high quality, large scale studies assessing outcomes of catheter-based embolectomy, with data derived from registries and pooled results, and with case series suggesting procedural success rates of 87% [14]. However, these studies may be subject to selection and publication bias. More recently, there have been several cohort studies and one RCT.

Although early studies were disappointing, the limitations of conventional therapy encouraged development of novel alternative treatments [15]. These approaches generally utilise local or directed thrombolysis intended to reduce thrombolytic dose, and mechanical thrombus disruption, including aspiration. Some techniques employ both. These methods remain in early clinical assessment.

The Ekos system uses ultrasound arrays within a sheath to deliver alteplase via acoustic streaming directly into the clot, thereby increasing the surface area of thrombus subject to contact with tPA. This may reduce the amount of thrombolytic required [16, 17]. Its use has been evaluated in several clinical studies.

The ULTIMA trial compared U-ACDT with intravenous heparin to intravenous heparin alone in 59 intermediate-risk patients, demonstrating improved RV/LV ratio at 24 h with U-ACDT and no significant difference in mortality or major bleeding at 90 days [18].

The SEATTLE II study also showed improved mean RV/LV ratio 48 h post procedure (1.55 vs 1.13; p < 0.0001) along with a reduction in mean PASP (51.4 mmHg vs. 36.9 mmHg; p < 0.0001) in 150 patients with acute massive, or submassive PE with no incidence of intracranial haemorrhage [19].

More recently, the OPTALYSE study was conducted on 101 haemodynamically stable patients with submassive PE, with four different treatment dose/duration protocols [20]. This showed a similar improvement in RV/LV ratio with each protocol, although there was a dose/duration-of-treatment relationship with thrombus burden reduction. There were two episodes of serious bleeding in the highest dose protocol (24 mg alteplase over 6 h), which was discontinued.

These three studies report on six different treatment protocols to use. It is currently unclear what the most appropriate dose/duration is, and it unlikely to be a “one size fits all” treatment, with protocol selection being patient dependent (clinical status, comorbidity, thrombus burden, bleed risk).

## Conclusion

There is a pressing need for high quality randomised control trials demonstrating improvement in robust clinical outcomes when comparing U-ACDT to simple anticoagulation in intermediate/high risk PE.

For massive PE, systemic thrombolysis can significantly reduce risk of death or cardiovascular collapse. However, these benefits are offset by a significant risk of bleeding, limiting its use in patients at risk of catastrophic haemorrhage.

The occurrence of massive PE in patients at high risk of haemorrhagic complications is likely to increase as the global population ages, and patients live with multiple comorbidities. This case series highlights the potential of catheter-based thrombolysis in clinical scenarios that are becoming more commonplace. The ability to achieve thrombus dissolution in the 3 patients discussed here proved lifesaving, and the application of U-ACDT in this high-risk group warrants comprehensive, prospective, and randomised evaluation.
